# Ceramide from sphingomyelin hydrolysis differentially mediates mitogen-activated protein kinases (MAPKs) activation following cerebral ischemia in rat hippocampal CA1 subregion^☆^

**DOI:** 10.1016/S1674-8301(10)60021-8

**Published:** 2010-03

**Authors:** Xian Sun, Chao Liu, Min Qian, Zhenghong Zhao, Jun Guo

**Affiliations:** a The Laboratory Center for Basic Medical Sciences, Nanjing Medical University, Nanjing 210029, China; b Department of Biochemistry and Molecular Biology, Nanjing Medical University, Nanjing, 210029, China

**Keywords:** ceramide, cerebral ischemia, extracellular-signal regulated kinase, c-Jun N-terminal protein kinase

## Abstract

**Objective:**

To explore the role that ceramide plays in the activation of mitogen-activated protein kinases (MAPKs) during cerebral ischemia and reperfusion.

**Methods:**

Rats were subjected to ischemia by the four-vessel occlusion (4-VO) method. The sphingomyelinase inhibitor TPCK was administered to the CA1 subregion of the rat hippocampus before inducing ischemia. Western blot was used to examine the activity of extracellular-signal regulated kinase (ERK) and c-Jun N-terminal protein kinase (JNK) using antibodies against ERK, JNK and diphosphorylated ERK and JNK.

**Results:**

At 1h reperfusion post-ischemia, JNK reached its peak activity while ERK was undergoing a sharp inactivation (*P* < 0.05). The level of diphosphorylated JNK was significantly reduced but the sharp inactivation of ERK was visibly reversed (*P* < 0.05) by the sphingomyelinase inhibitor.

**Conclusion:**

The ceramide signaling pathway is up-regulated through sphingomyelin hydrolysis in brain ischemia, promoting JNK activation and suppressing ERK activation, culminating in the ischemic lesion.

## INTRODUCTION

Mitogen-activated protein kinases (MAPKs) are evolutionarily highly conserved serine/threonine protein enzymes that participate in signal transduction pathways connecting cell surface receptors to key regulatory nuclear and other intracellular targets[1],[2]. The MAPK cascade is composed of at least three sequential intracellular protein kinase activation steps initiated by the activation of MAPK kinase kinase (MAPKKK, MEKK), which in turn activates MAPK kinase (MAPKK, MKK or MEK) and finally MAPK[1],[2]. Members of the MAPK family, include extracellular signal-regulated kinases (ERK), c-jun-N-terminal kinases (JNK) and p38 MAPK, play an important role in neuronal survival/damage in response to cerebral ischemia[3]. JNK and p38 MAPK are regarded as stress-activated protein kinases (SAPK) and are important in apoptosis and inflammation[4]. ERK can be activated through a Ras/Raf/MEK/ERK cascade, promoting a spectrum of cellular responses, including growth and differentiation, as well as survival[5].

Many signal molecules or messengers released following brain ischemia contribute to the activation of MAPKs. Calcium has been suggested to be a key signal in cerebral ischemia[3]; ceramide, one of the products induced by ischemia and a second messenger, mediating non-calcium-induced signaling cascades[6], is also assumed to have great importance for cerebral ischemic lesions[7],[8]. It is known that ceramide is mainly generated from the hydrolysis of sphingomyelin (SM) through the action of sphingomyelinase as a bypass product of the SM pathway[9],[10]. Ceramide has been reported to participate in apoptosis and directly or indirectly target a number of enzymes and signaling components, including proline-directed kinases such as the ceramide-activated kinase (CAPK) or JNK, the protein kinase c ζ (PKC ζ), ceramide-activated protein phosphatase (CAPP), phospholipases such as cPLA2 or PLD, transcription factors such as NF-κB, and CPP32-like caspases[10]–[12].

In this study, we concentrated on the association between ceramide production and activation of JNK and ERK following cerebral ischemia. The data indicate that 1 h ischemia/reperfusion results in JNK activation and ERK inactivation. Moreover, a sphingomyelinase inhibitor inhibited the JNK pathway and promoted ERK activation. Thus, ceramide from sphingomyelin hydrolysis differentially mediates MAPKs activation following cerebral ischemia and reperfusion in rat hippocampus.

## MATERIALS AND METHODS

### Animal model

Animal surgery was approved by the Institutional Animal Care and Use Committee and conformed to international guidelines for the ethical use of animals. Efforts were made to minimize the number of animals used and their suffering. Adult male Sprague-Dawley rats weighing 250-300 g were obtained from Experimental Animal Center of Nanjing Medical University. Rats were housed in air-conditioned cages with free access to food and water. Cerebral ischemia was induced by the four-vessel occlusion method as described previously[6]. In brief, animals were anesthetized with 20% chloral hydrate (300 mg/kg, i.p.). Both vertebral arteries were occluded by electrocauterization and meanwhile silk threads were placed around both common carotid arteries without interrupting the blood flow. Twenty-four hours later, the bilateral carotid arteries were precisely occluded with aneurysm clips for 10 min, after which the clips were removed and the rat was allowed to recover. To minimize variability, the following criteria were met: ① loss of corneal reflex, ② bilateral pupil dilation during the entire ischemic period, ③ completely flat electroencephalogram, ④ rigor of the extremities and vertebral column and ⑤ body temperature was maintained at approximately 37°C.

### I.C.V. infusion and administration of drugs

The sphingomyelinase inhibitor N-tosyl-l-phenylalanine chloromethyl ketone (TPCK; 10 µg/µl in DMSO, 3 µl; Sigma-Aldrich Co., St Louis ,MO, USA) or the same volume of vehicle was injected into the cerebral ventricle (0.8 mm posterior and 1.5 mm lateral to the bregma; 3.5 mm deep) by microinjector 30 minutes before inducing the ischemia.. The injector was kept in place for an additional 5 min after the injection so as to minimize any possible backflow of the liquid along with the injection void.

### Western blot

All the rodents underwent 4-VO, endured 10-min ischemia, and were sacrificed by decapitation at different time points after different periods of reperfusion (10 min, 1 h, and 6 h). The hippocampi were quickly separated on ice and the hippocampal CA1 subfields were dissected according to previously published procedures[13]. The separated tissues were then homogenized in 1:10 (weight/volume) chilled homogenization buffer A [50 mM HEPES (pH 7.4), 100 mM KCL, 1 mM Na_3_VO_4_, 50 mM NaF, and 1 mM PMSF] containing 1 % mammalian proteinase inhibitor cocktail (Sigma-Aldrich Co.). Proteins in the cytoplasm and membrane were obtained after centrifugation at 800 g for 10 min at 4°C. The supernatant samples were extracted and stored at -80°C until assayed. Protein extracts were denatured by boiling for 5 min in sample loading buffer.

Denatured samples were separated by 10 % SDS-polyacrylamide gels and then semi-electrotransferred onto nitrocellulose filter (NC, pore size, 0.2 µm). The transferred proteins were reacted respectively with primary antibodies against ERK (1:5,000 dilution), against diphosphorylated ERK (1:2,000), against JNK (1:5,000), and against diphosphorylated JNK (1:1,000) for 4 hours at room temperature. The resulting immune complexes were then reacted with alkaline phosphatase conjugated secondary goat anti- rabbit IgG (1:10,000). All antibodies were purchased from Cell Signaling Technology (Beverly, MA, USA). Detection was developed by enhanced chemiluminescence (Amersham Bioscience, Piscataway, NJ, USA). The bands on the membranes were scanned and analyzed by an image analyzer.

### Statistical analysis

Data are expressed as means±standard deviation (SD) from at least three independent animals. Statistical analyses was performed using one-way ANOVA followed by the Student-Newman-Keuls(SNK) method for protein expression levels. P values of less than 0.05 were considered significant. All analyses were performed using the SPSS software (Version 13.0, SPSS Inc., USA).

## RESULTS

### Reperfusion following ischemia leads to temporal alteration of p-ERK and p-JNK in the rat hippocampal CA1 subregion

Rats in experimental groups were subjected to ischemia for 10 min followed by reperfusion for 10 min, 1h or 6 h, while rats in the sham group were only subjected to identical anesthesia and surgery without ischemia and reperfusion. To reveal the changes in ERK and JNK activity in the hippocampal CA1 subregion of rats for different durations of reperfusion (10 min, 1 h and 6 h), we used a specific antibody against ERK phosphorylation at Thr202/Tyr204 and an antibody against diphosphorylated JNK at Thr183/Tyr185. Western blot analyses were conducted to detect p-ERK, total ERK, p-JNK and total JNK levels.

**Fig. 1:**
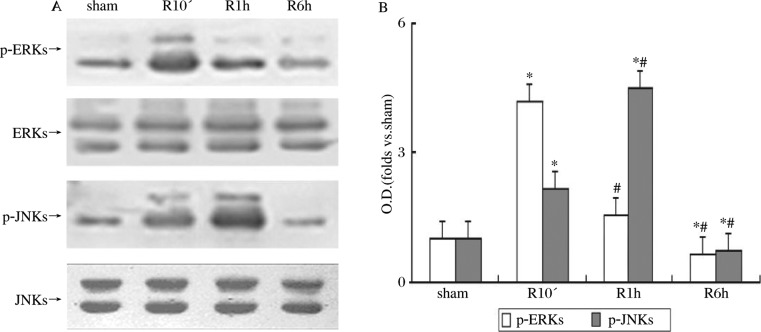
ERK and JNK activity assay at different reperfusion times following ischemia in the hippocampal CA1 subregion of rats. Samples were obtained from rat hippocampal CA1 subfield of sham control animals and animals that underwent 10 min, 1 h and 6 h reperfusion following 10-min cerebral ischemia. Proteins were measured using antibodies against p-ERK (Thr202/Tyr204) and ERK, and against p-JNK (Thr183/Tyr185) and JNK. A: western blot analysis of content and phosphorylation of ERK and JNK of samples after 10-min, 1-h and 6-h reperfusion following 10-min ischemia. B: quantitative determination results of ERKs and JNKs phosphorylation. Optical density (OD) data are presented as means±SD (*n* = 3) and expressed as the magnitude of the alteration compared to the sham control. *Significantly different (*P* < 0.05) from the sham control; ^#^Significantly different (*P* < 0.05) from the respective 10-min reperfusion groups.

As shown in ***Fig. 1***, ERK activity rapidly decreased following a strong activation at 10-min reperfusion after ischemia compared with sham-operated rats (*P* < 0.05). However, the p-JNK level gradually increased (*P* < 0.05) and reached its peak at 1h reperfusion post-ischemia (*P* < 0.05). The levels of the two phosphorylated proteins began decreasing as the reperfusion period was prolonged (*P* < 0.05).

The changes in p-ERK and p-JNK arose from phosphorylation, because there was no alteration of the amount of ERK protein and JNK protein in each group in response to different treatments (*P* > 0.05; ***Fig. 1***).

### Effects of TPCK on ischemia-induced activation of ERK and JNK

**Fig. 2:**
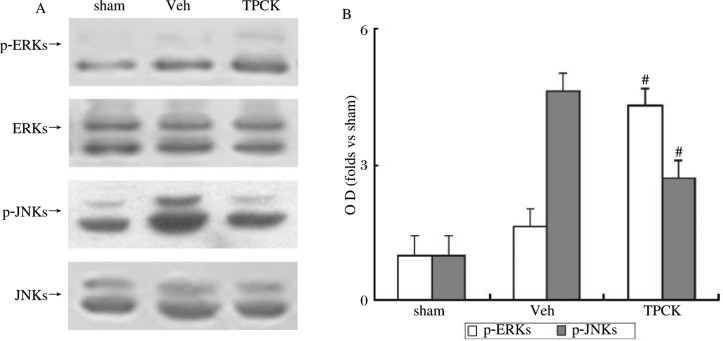
Effect of TPRK on diphosphorylation of ERK and JNK in rat hippocampal CA1 subregion. Samples were obtained from hippocampal CA1 subregion of rats subjected to 1-h reperfusion following 10-min ischemia after administration of TPCK (TPCK, i.c.v) or the same dose of vehicle (Veh). A: western blot assay of p-ERK and p-JNK with and without TPCK during 1-h reperfusions following 10-min ischemia. B: quantitative representation of ERKs and JNKs phosphorylation. OD data are presented as means±SD (*n* = 3) and expressed as magnitude of the alteration compared to the sham control. ^#^Significantly different (*P* < 0.05) from respective vehicle-treated group.

TPCK, a cell-permeable serine protease inhibitor, can attenuate the generation of ceramide via inhibiting sphingomyelinase[14]. TPCK or the same dose of vehicle was administered 30 minutes before inducing ischemia. After 1 h reperfusion, compared with the vehicle-treated rats, rats treated with TPCK exhibited a significant increase in the level of p-ERK (*P* < 0.05), but a decrease in the level of p-JNK (*P* < 0.05). The disparity between p-ERK and p-JNK resulted from phosphorylation, as no alteration of the total ERK or JNK protein was detected during treatment (*P* > 0.05; ***Fig. 2***).

Together, these data suggest that rapid accumulation of ceramide probably results from cleavage of sphingomyelin induced by activated sphingomyelinase and is responsible for the activation of JNK and a sharp inactivation of ERK after cerebral ischemia and reperfusion.

## DISCUSSION

Cerebral ischemia/reperfusion produces multiple changes in signaling cascades that are crucial for cell survival/damage. A detailed understanding of these changes can provide not only fundamental insights but also potential targets for therapeutic intervention. In this study, aimed at a better understanding of the activation of MAPK, we investigated the role that ceramide plays in the activation of MAPK during cerebral ischemia and reperfusion.

Consistent with previous studies, JNK, which contributes to neuronal apoptosis, exhibited a peak of activation at approximately 1 h of reperfusion[15], while ERK was undergoing a sharp inactivation following the early intense activation that was elicited by cerebral ischemia. The JNK pathway can be intensely activated by factors such as TNF-α and IL-1β, which are known to be increased after a stroke and have been shown to be involved in the mechanisms underlying ischemia-induced neuron apoptosis[16]. ERK activation has been described as being calcium-dependent[17],[18], and Ca^2+^ signals might effectively stimulate calcium-dependent kinases like CaMK II, Src, PYK2 and PKC that are mainly implicated in ERK activation as upstream regulatory molecules[19]. Cerebral ischemia and resulting massive calcium influx could induce the biphasic activation of ERK in rat hippocampus[20],[21], but it has been demonstrated that the rapid inactivation of ERK following the early intense increase occurred in a calcium-independent manner because inhibition of the calcium influx through NMDA receptor could not relieve the inactivation of ERK[21]. Therefore, some other mechanisms may account for this effect.

In order to better understand the different changes of JNK and ERK at 1h reperfusion, the cell-permeable serine protease inhibitor, TPCK was used. With TPCK pretreatment, JNK activity decreased dramatically while the sharp inactivation of ERK was visibly reversed, indicating that the ceramide signaling pathway activated by sphingomyelin hydrolysis led to activation of JNK but inactivation of ERK. Based on current knowledge, we considered the possibility that ceramide induces apoptosis through activation of downstream effectors such as the JNK pathway[12],[22], so a low level of p-JNK is not unreasonable since ceramide generation is blocked by the sphingomyelinase inhibitor TPCK. Nevertheless it is still an open question as to how ceramide activates the JNK pathway. It has been suggested that some intermediate enzymes described in other cellular systems may be implicated with this process in neurons, such as the small G-protein Rac-1[23], the MAP kinase kinase kinase TGFb-activating kinase 1(TAK1)[24], PKC ζ [25], the Apoptosis Signal regulated Kinase 1 (ASK1)[26] or the MAP three kinase 1 (MTK1/MEKK4) [27].

In contrast to JNK, ceramide has a negative impact on the ERK pathway as mentioned above. The molecular mechanism by which ceramide inhibits the ERK pathway in neurons remains unclear. As is known, ERK activity depends on its phosphorylation state, resulting from the balanced action of both ERK kinases and ERK-directed protein phosphatases. The immediate upstream kinase of ERK, MEK, is also activated by phosphorylation through kinases in the Raf serine/threonine kinase family[2]. PP2A, a heterotrimeric holoenzyme, has been identified to be the major ERK phosphatase which is responsible for ERK inactivation in ischemic brain tissues[28]. Further, PP2A was reported to be a ceramide-dependent phosphatase[29] and its activity increases through interaction with ceramide on the catalytic subunit[12]. In this way an elevation of ceramide encourages PP2A to depress the activity of ERK. In addition, ceramide could directly target the CAPK or PKC ζ. The CAPK, which has been hypothesized to be the kinase suppressor of Ras (KSR)[30], is probably another negative regulator of the ERK pathway in response to ceramide. It has also been reported that ceramide could act on MLK3/SPRK that functions as a MAPKKK in the stress-activated JNK pathway, and contribute to inhibition of the ERK pathway through sustained JNK activation[31]. Thus, ceramide participates in neuron injury partially through ERK inactivation following cerebral ischemia.

In summary, ceramide derived from sphingomyelin hydrolysis strongly promotes JNK activation and suppresses highly activated ERK during the early period of cerebral ischemia and reperfusion, which in turn, attenuates neuroprotective effects and results in ischemic lesions. Additional investigations are required to fully illuminate the molecular mechanisms by which ceramide functions during cerebral ischemia and reperfusion.
